# A controlled trial of mental illness related stigma training for medical students

**DOI:** 10.1186/1472-6920-11-51

**Published:** 2011-07-29

**Authors:** Aliya Kassam, Nick Glozier, Morven Leese, Joanne Loughran, Graham Thornicroft

**Affiliations:** 1 Section of Community Mental Health Health Service and Population Research Department, King's College London, Institute of Psychiatry, De Crespigny Park, Denmark Hill, London SE5 8AF UK; 2 Department of Community Health Sciences University of Calgary 3rd Floor TRW 3280 Hospital Drive NW, Calgary, T2N 4Z6 Canada; 3 Discipline of Psychiatry, Sydney Medical School, The University of Sydney, NSW 2006 Australia; 4 Mental Health Promotion, Rethink, Floor 15, 89 Albert Embankment, London, SE1 7TP UK

## Abstract

**Background:**

The evidence base for mental illness related stigma interventions in health care professionals and trainees is underdeveloped. This study aimed to examine the impact of mental illness related stigma training on third year medical students' knowledge, attitudes and behaviour related to people with mental illness.

**Methods:**

A non-randomised controlled trial was conducted with 110 third year medical students at a medical school in England to determine the effectiveness of a mental illness related stigma training package that targeted their knowledge, attitudes and behaviour.

**Results:**

We detected a significant positive effect of factual content and personal testimonies training upon an improvement in knowledge, F(1, 61) = 16.3, p = 0.0002. No such difference was determined with attitudes or for behaviour.

**Conclusions:**

Knowledge, attitudes and behaviour may need to be separately targeted in stigma reduction interventions, and separately assessed. The inter-relationships between these components in mental health promotion and medical education warrant further research. The study next needs to be replicated with larger, representative samples using appropriate evaluation instruments. More intensive training for medical students may also be required.

## Background

The classical definition of stigma is 'a trait that is deeply discrediting that reduces the barer from a whole to a tainted, discounted one' [1]. Previous models of stigma however have had little connection to disability policy or clinical practice [2]. The concepts of knowledge, attitudes and behaviour, that are relevant to both medical education [3,4] and health promotion [5,6] can be applied to the stigma of mental illness [2]. Problems pertaining to a lack of knowledge constitute ignorance, and problems in attitudes can be seen as prejudice, while problems in behaviour may be considered as discrimination. In fact service users often report difficulties with how healthcare staff treat them such as having negative attitudes towards people with mental illness, and by providing a lower standard of care [7-10].

Like the general public, medical students often hold the stereotypical views that people with mental illness are unlikely recover and people with severe mental illness are dangerous and violent [11,12]. It has been reported that 28% of medical students thought people with mental illness 'are not easy to like' and this rose to 56% at 2 year follow-up. One study found no differences between medical and nursing undergraduates regarding their attitudes towards people with schizophrenia [13]. What is notable is that 50% of these medical and nursing students thought that people with schizophrenia would never recover and 78% considered people with schizophrenia to be dangerous and violent. Furthermore 95% did not feel they had enough information about schizophrenia [13]. People with mental illness also have poorer physical health, in part because of 'diagnostic overshadowing' [14-16] in which the physical problems of a patient are "over-shadowed" and attributed to their psychiatric diagnosis [16].

There is growing evidence that mental illness related stigma can be reduced. Social contact has been shown to be effective in decreasing negative attitudes about mental illness [17-19], yet the impact of the psychiatric clerkship in the medical school curriculum has shown mixed effects in terms of both favourable and negative changes in medical students' attitudes [20-22]. This may be due to mainly seeing people on the ward who are very unwell [20-22]. Social contact with a person who has mental illness has been shown to be an effective stigma reducing intervention in police officers and young people [18-22]. Attempts to use a behavioural component such as communication skills in role-play training in mental illness related stigma however are rare. One study examined the effect of role-play training on attitudes which showed no effect [23] while another study showed that self-directed role play activity had an effect on attitudes towards depression [24]. Neither study measured the effect of such training on the behaviour of medical students.

The majority of studies of mental illness related stigma training in medical students have used either a lecture delivered by a professional or a non-interactive video and used evaluation measures that conflated knowledge and attitudes [25-27]. One study however investigated didactic teaching versus combined didactic teaching with self-directed learning using targeted knowledge and attitude evaluation instruments. The self-directed learning component incorporated creative or artistic methods to express their understanding of depression. The didactic teaching in addition to the self-directed learning showed greater improvements in knowledge about depression and improvements in attitudes about depression [24].

Regarding the measurement of attitudes, just as there are scales assessing medical students' attitudes towards people with mental illness before and after a psychiatric clerkship, scales assessing medical students' attitudes towards specialising in the field of psychiatry have also been developed. This is because there is stigma by association; that is, because the field of psychiatry is responsible for treating people with mental illness, the field itself also becomes stigmatised. This may lead to psychiatry being a less favoured potential career choice due to such negative attitudes [28].

The Attitudes Towards Psychiatry (ATP-30) scale [29] has been used in previous medical student research. In one study, a significantly negative change was detected in attitudes of first-year medical students on an anatomy course as well as with third year paediatric students. There was a significantly positive change in attitudes towards psychiatry among third and fourth-year medical students, but not occupational therapy students who were exposed to clinical work with patients who had a mental illness [29].

Another study showed no significant differences between the pre- and post- attitudinal scores on the ATP-30 with regards to the sixth-year medical students who completed the practical psychiatry training [30]. Given these inconsistencies and that the primary focus of this study was medical students' attitudes towards people with mental illness rather than Psychiatry; we chose not to use this scale. Nevertheless, it must be emphasized that the ATP-30 does have relevant items that could be used to determine whether didactic teaching and role play can change knowledge attitudes and behaviour.

In this study we undertook a controlled trial to compare the effects of 3 different interventions, and directly assessed students' mental illness related knowledge, attitudes and behaviour towards people with mental illness.

## Methods

### Aims

This study aimed to examine the impact of mental illness related stigma training on third year medical students' knowledge, attitudes and behaviour related to people with mental illness.

### Study design

The study was a non-randomised controlled trial with three conditions:

A. Control Condition (CC): none of the intervention elements below.

B. Experimental Condition 1 (EC1): A presentation on mental illness related stigma which included the social and personal impacts of stigma against people with mental illness together with personal testimonies from a mental health service user and a caregiver of a person with mental illness.

C. Experimental Condition 2 (EC2): As B above plus a role-play training session in a class room setting with mental health service user and caregiver feedback.

We hypothesised that there would be greater, more favourable change in the knowledge, attitudes and behaviour of the experimental conditions (EC1 and EC2) combined, compared to the control condition (CC). Furthermore, a greater change would be detected in the intervention condition that had the factual content, personal testimonies and role-play training compared to the condition with just the factual content and personal testimonies alone.

### Sample

Students were recruited before the beginning of their third year of medicine, which began with a two week introductory course. All students were given the same standard training during this two-week course by the medical school. Material relating to psychiatry and mental illness during this course was minimal and consisted of a brief introduction without any contact from service users or caregivers. Third year medical students were chosen because at this particular medical school their psychiatry rotation/clerkship took place in the third year hence we did not want them to have had clinical contact with patients which could confound the results.

Students were recruited by e-mail. Each student was sent an email introducing the study and what it would entail if they chose to participate. A brief presentation was also given to students at the start of the two-week course telling them about the study. Students were told that their participation in the study was entirely voluntary and their grades would not be affected in any way if they chose not to participate. Students who chose to participate were told that their responses would be kept anonymous and would not affect their grades or course work.

At the end of the academic year, data pertaining to the Psychiatry assessment grades of participants and non-participants were obtained from the registrar of the University to which the medical school belonged. Global scores on the medical school Psychiatry assessments of the participants and non-participants were used to estimate the representativeness of the sample.

### Inclusion/exclusion criteria

Students were included in the trial if they were registered for their third year of medicine at the medical school and they: (i) had a valid e-mail address that was on the list provided by the registrar to the researcher; (ii) were attending the two week introductory session. Participants were recruited two weeks prior to the intervention. The recruitment period was short because the introductory timetable and list of registered students was not finalised until the start of the two week introductory period. Students began their psychiatry rotation immediately after the two week introductory period.

### Allocation

Allocation into the trial conditions was done by an administrator at the medical school blind to the proposed intervention and independent from the research team. Each trial condition consisted of clusters of firms which were put together pseudo-randomly by the office of the registrar. This meant that student groups of six to ten students were placed together for learning purposes within the medical school curriculum and remained together throughout the year. The basis for allocation was whether the clusters of firms had space in their timetable at the same time when the experimental condition could take place due to logistical issues at the medical school. The nature of allocation of students by the medical school administration, allowing for the preferences of individual students, was not compatible with randomisation.

For the evaluation component of knowledge and attitudes, students were contacted by e-mail. For the evaluation component of behaviour, a role-play assessment of communication skills was allocated by the administrator to a subset from each of the conditions (CC, EC1, EC2) to be examined when they had spaces in their timetable.

### Training intervention

The training intervention was developed separately from the evaluation team and was developed by a mental health charity. The interventions were designed to address the knowledge, attitudes and behaviour model with the factual component targeting knowledge, personal testimonies from service users and caregivers targeting attitudes and role-play training targeting behaviour. The intervention in EC1 consisted of a 1-hour time slot including: (i) a 15 minute factual component presentation from the mental health promotion officer of the charity on the social impacts of stigma and discrimination against people with mental illness including themes such as healthcare, employment, civil society participation, personal relationships, medication, media and aggressive behaviour; (ii) a 15 minute personal testimony from a mental health service user who was taking medication for schizoaffective disorder describing their personal perspectives and experiences of having a mental illness, stigma and discrimination; and (iii) a 15 minute personal testimony from a caregiver of a person with schizophrenia discussing their personal perspectives and experiences of caring for a person with a mental illness, stigma and discrimination; (iv) a 10 minute question and answer session between the students and the mental service user and caregiver; (v) a 5 minute allotment to complete a short satisfaction questionnaire about the entire lecture (factual component and personal testimonies).

The additional intervention for EC2 was also developed by the charity. This consisted of two 10-minute role-play scenarios (a parent and daughter seeking help for their daughter's mental health problems and a service user who had a physical health complaint). Role-players were recruited from the role-player company used by the medical school and were instructed about the scenario by the researcher. Students were divided into groups of 8 to 10 and systematically allocated to different classrooms. A facilitator and either a service user or caregiver observer were present among the students. A student was asked to volunteer for the first role-play scenario and the role-players were asked to enter the room by the facilitator. After the role-play, the medical students gave their feedback on how they felt they performed and what they found challenging. This was followed by the role-players, observing service user or caregiver and fellow students sharing their feedback regarding the communication skills used by the medical student with the role-player(s) in the scenario. This process was repeated for the second role-play with a different student volunteer. A satisfaction questionnaire was completed after the role-play training.

### Assessments

At baseline the students were asked a number of demographic factors, social contact of someone with mental illness and intended area of specialisation after medical school and had their knowledge and attitudes towards mental illness evaluated. Students were asked to complete the assessments online two weeks before the intervention (baseline) and were sent reminder e-mails from the researcher. On the day of, but prior to, the intervention, students also had the opportunity of completing the instruments in hardcopy. One week after all of the interventions and role-play assessments were completed, students were reassessed using baseline measures, either online or through hard copy. Primary knowledge and attitude outcome data were obtained from the Knowledge Quiz and the Mental Illness: Clinicians' Attitudes (MICA) scale [31]. The secondary outcome measure was a role-play assessment carried out by the role-players of which there were no baseline data.

### Measures

a) Knowledge: The Knowledge Quiz was designed by the study team to assess knowledge regarding information on stigma and discrimination against people with mental illness. This consists of 10 true or false items based on key information from current literature and legislation on areas of prevalence, violence, serving the community, media, employment, black minority ethnic (BME) issues, access to healthcare, cost of mental healthcare and the importance of social networks for people affected by mental illness. Items generated for the Knowledge Quiz were reviewed by key researchers in the field of stigma and mental illness. Although not formally validated it has both face and content validity with items derived from sources related to stigma and discrimination in people with mental illness. The knowledge quiz is scored in a manner in which a high total score represents more knowledge about people with mental illness, stigma and discrimination.

b) Attitudes: Mental Illness: Clinicians' Attitudes (MICA) scale The development and validation of the MICA scale has been described elsewhere [31]. It is comprised of 16 items with a (1-6) Likert scale. A low total score (16 minimum) represents less stigmatising attitudes towards mental illness and psychiatry. The maximum score on the MICA is 96 showing very stigmatising attitudes. The MICA scale has satisfactory internal consistency with a Cronbach's alpha coefficient of 0.79 and a test-retest reliability of 0.80.

We felt that meaningful change in knowledge and attitudes corresponds to at least 10% change in the total mean score although this is somewhat arbitrary given the nature of this study which was exploratory. In order to detect a 10% change in the total MICA scale scores before and after the intervention with 80% power using a two-tailed test, a minimum of 31 students would be required at an alpha level of 0.01.

c) Role Play Behaviour: Role play does not necessarily equate to behaviour. However in medical education, role-play is often used as a teaching method that is valued by students in the acquisition of communication skills. Furthermore, role play promotes active learning [32]. The role play assessment constituted a list of fifteen items pertaining to behaviours (both verbal and non verbal) that could be interpreted as discriminating by a person with mental illness or their caregiver. Each of these items is scored either with a 0, 1 or 2; a higher score indicating more positive communication behaviour and comprised the component score. There was also a global score for the checklist which was used to indicate the rater's overall impression of how the student performed. The global score categories were Excellent = 5, Very good, 4 = Good, 3 = Pass, 2 = Borderline Fail, 1 = Fail. The development and validation of the role-play assessment was based on an actual observed structured clinical examination (OSCE) station checklist, tailored to issues of stigma with input from service users and caregivers and is described elsewhere [33].

### Statistical analysis

Analyses were carried out using only data from students who completed instruments at both baseline and post-intervention. Non-completer data were compared to completers to assess bias. Baseline scores were compared between the arms of the trial, and an analyses of covariance (ANCOVA) controlling for baseline scores, gender, and psychiatry assessment grades (from the medical school at the end of the academic year) was conducted to estimate differences across the three conditions regarding knowledge and attitudes. For behaviour, only a post intervention assessment was available. Analysis was carried out using regression in STATA version 10. Residuals from the regressions were examined for normality. Two pre-specified contrasts were tested: 1) to compare the two interventions combined with the control condition, and 2) to compare the two intervention arms with each other.

### Ethics

This project was approved by the Local Research Ethics Committee of Bexley and Greenwich (06/Q0707/56) for the National Health Service in the UK.

## Results

**Figure 1:**
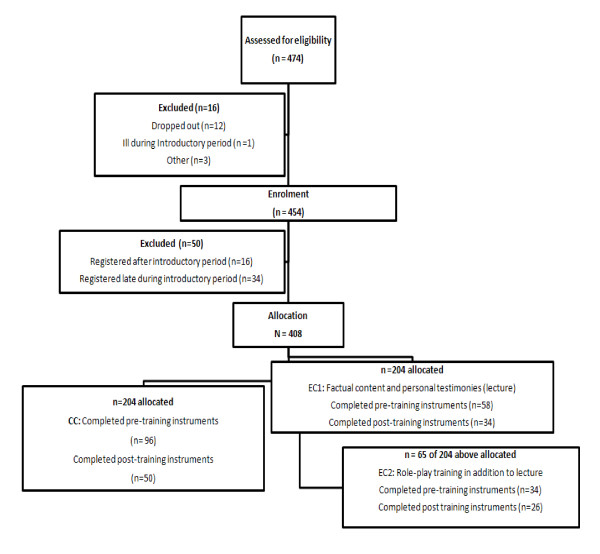
**Recruitment and follow-up for study**. (Note: Baseline = 96+ 58 +34 = 188 Follow-up = 50 + 34 + 26 = 110).

Figure 1 shows how the eligibility of the students was determined from information given by the Medical School Registrar. The response rates for the primary outcome instruments are also provided. Of the 408 eligible medical students, 211 students responded (52% response rate). However data from 23 were not used because there were more than 20% of items missing at baseline and 17 had completed baseline instruments online after the intervention had taken place as the online deadline had passed for baseline data collection. Of the 188 students who completed baseline instruments 110 (59%) had both pre- and post intervention instruments (Knowledge Quiz and MICA scale) completed and were used for subsequent analyses. Of the 204 allocated to EC1, 154 attended the lecture and completed satisfaction questionnaires. Of the 65 of the 204 allocated to EC2, 33 attended the role-play training and completed satisfaction questionnaires.

The demographic information for the students is shown in table 1. The majority of the students taking part were female, of white ethnicity and unsure of what area of medicine they would specialise in. Nearly half of the sample personally knew someone with mental illness. No other data were available for the students.

**Table 1:** Demographic information for participants in trial

	Control Condition (n = 50)	Lecture only (n = 34)	Lecture plus role play (n = 26)
**Mean age in years (sd)**	22.9 (3.3)	22.4 (2.5)	22.8 (4.4)

	N (%)	N (%)	N (%)

**Male**	18 (36)	9 (27)	2 (8)

**Ethnicity**			

**White**	28 (56)	21 (62)	13 (50)

**Black**	-	-	2 (8)

**Asian**	14 (28)	2 (6)	5 (19)

**Chinese**	3 (6)	9 (26)	3 (11)

**Mixed**	3 (6)	2 (6)	2 (8)

**Other**	2 (4)	-	1 (4)

**Intended speciality**			

**Surgery**	10 (20)	4 (12)	3 (11)

**General Medicine**	4 (8)	4 (12)	3 (11)

**Paediatrics**	9 (18)	6 (18)	1 (4)

**Gynaecology/Obstetrics**	3 (5)	3 (9)	2 (8)

**Psychiatry**	-	2 (6)	1 (4)

**Laboratory Medicine**	-	-	1 (4)

**Family Medicine**	3 (5)	2 (6)	-

**Public Health**	-	-	1 (4)

**Other**	1 (4)	1 (3)	1 (4)

**Unsure**	20 (40)	12 (35)	13 (50)

**Personally knows someone with mental illness**	27 (58)	19 (53)	17 (54)

There was a significant difference between the grades, with the group of medical students who participated in the study having higher psychiatry assessment scores than non-participants, 4.3 vs. 4.1, p = 0.008. This shows that those medical students who chose to take part in the study either by way of completing baseline instruments, or participating in the intervention conditions had medical school psychiatry assessment grades that were significantly higher (more favorable) than those students who did not participate.

Table 2 shows the mean scores on the Knowledge Quiz and attitudes before and after the intervention, and Table 3 shows the adjusted differences between each of the intervention conditions compared to control at follow up, controlling for baseline, gender, personally knowing someone with mental illness and medical school psychiatry assessment grades.

**Table 2:** Knowledge and attitude scores for each trial arm.

	**Control Condition (n = 50)** **Mean (sd)**		**Lecture only** **(n = 34)** **Mean (sd)**		**Lecture plus role-play** **(n = 26)** **Mean (sd)**	
	
**Total score**	**Before**	**After**	**Before**	**After**	**Before**	**After**
	
**Knowledge**	7.1 (1.5)	7.2 (1.5)	7.0 (1.4)	8.1 (1.2)	7.3 (1.4)	8.6 (1.1)
	
**Attitudes**	38.3 (7.2)	38.0 (7.2)	37.4 (5.8)	36.3 (5.7)	40.3 (8.5)	38.6 (7.9)

**Table 3:** Comparisons of trial arms (knowledge and attitudes) adjusting for baseline scores^1^

a) Knowledge	Adjusted difference	95% CI	p
Interventions (combined) vs. Control	-1.192	-1.756 to -0.627	< 0.001

Lecture vs. Lecture plus role play	-0.432	-1.213 to -0.348	0.274

**b) Attitudes**	**Adjusted difference**	**95% CI**	**p**

Interventions (combined) vs. Control	-1.569	-3.349 to 0.219	0.085

Lecture vs. Lecture plus role play	-0.341	-2.828 to 2.136	0.785

^1 ^gender, medical school psychiatry assessment grades, and personal knowledge of someone with mental illness

### Knowledge

There were no significant differences in total scores on the Knowledge Quiz at baseline among the control condition, experimental condition 1 and experimental condition 2 (p = 0.668). At follow up there was a significant favourable effect of training on knowledge about people with mental illness and stigma with an adjusted mean difference of 1.19 (95%CI 0.63-1.76) (comparing the two interventions combined with control), but no evidence for a difference between the two different types of intervention).

Regarding knowledge, the Knowledge Quiz was developed solely for the purpose of this study; previous data had not been collected in order to compute a power calculation. A post-hoc power calculation however shows that our study had satisfactory power as we had 80% power to detect a 15.7% change in Knowledge Quiz scores using a two-tailed test in a sample size of 34 students at an alpha level of 0.01.

### Attitudes

There was no significant difference between the three trial arms for attitudes at baseline (p = 0.307). There was weak evidence (p = 0.085) that any intervention condition led to less stigmatising scores than the control condition see Table 3. This was despite achieving adequate samples in the CC and EC1 conditions.

### Behaviour

In total, 68 students consented to take part in the role-play assessments, 22 of whom were from CC, 21 of whom were from EC1 and 25 of whom were from EC2. The means (sd) for the global role play scores for CC, EC1 and EC2 were 3.14 (0.77) 3.52 (0.87) 3.52 (0.77) respectively. The contrast of the combined experimental conditions (EC1 and EC2) with the CC was significant at a borderline significance level, at p = 0.069, but there was no evidence for a difference between the two intervention conditions.

There were no differences in overall scores when comparing the component scores of the service user scenario with the caregiver scenario or when comparing the global scores of the service user scenario with the caregiver scenario.

### Overall satisfaction with training

Over 80% of the students in the EC1 and EC2 conditions reported that the material in the lecture was clear, pitched correctly, of relevant content to their medical education and helped them develop a better understanding of mental illness related stigma. When students were asked about what they liked best about the lecture, 85% of students reported the personal testimonies from the service user and caregiver. Some comments were:

'Patient and caregiver inputs - more useful than text books/consultant viewpoints.'

'Having people present their own experiences - especially someone who suffers from a mental health problem - an excellent way to prove that these people should not be discriminated against and are actually very capable.'

'Hearing from someone with a mental illness that I considered really serious and long lasting and seeing that he is now just like any other "normal" person.'

Over 70% of the 34 students students who completed satisfaction questionnaires after the role-play training reported that the service user and caregiver feedback regarding the students' performance during a history taking was useful and over 80% of students reported that they felt more confident in speaking to a person with mental illness or a caregiver of a person with mental illness. When students were asked about what they liked best about the role-play training, students reported the service user and caregiver feedback, the opportunity to practice and learn from the difficult situations raised in the role-plays. Relevant comments included:

*'I feel less intimidated at the prospect of interviewing a patient in these situations'*.

'I think my behaviour would be warm and welcoming as before but I do feel that I've learnt how to speak to people with mental illness in a better way.'

'An opportunity to explore issues which may arise in this scenario. Feedback very productive and useful.'

'I already had the attitude that patients with mental illness required great respect. I still feel this way and the role-play reinforced this.'

## Discussion

The aim of this study was to evaluate (i) whether some kind of intervention would be effective in changing knowledge, attitudes and behaviour compared to the usual medical school curriculum, and (ii) whether the addition of user and carer enabled role-play training to a lecture with a factual component with service user and caregiver personal testimonies was more effective than the lecture alone.

The lecture sessions, with or without the role playing, were successful in increasing knowledge in a favourable direction but there was little evidence that the role-play training provided additional impact to the lecture. There was some weak evidence for the effectiveness of the combined intervention condition in improving attitudes and behaviour, but again no evidence for an additional effect of the role play intervention.

Regarding how representative the sample recruited was to the rest of the students, there were significant differences in those medical students who participated in the study compared to those that did not. This was expected as participants who would be more interested in the topic of mental illness related stigma were more likely to have participated and thus likely to take more of an interest in psychiatry thereby performing better at it overall.

### Methodological limitations

The study was not randomised as students were allocated to firms by the medical school Registrar and this determined whether or not they would be given the intervention since certain clusters of firms had space in their two week introductory session timetable whereas others did not. There were two stages at which selection bais may have occurred. First our participants showed a gender bias in that female students were over-represented. Second, the proportion followed up was also low. While we controlled for some factors associated with loss to follow up, we were not able to characterise these completely.

A number of factors may have contributed to the low follow up rate. We were allowed only one hour for the lecture intervention with personal perspectives and one half hour for the role-play training intervention by the medical school so the instruments were not administered within these session times. Students were e-mailed requesting them to complete the instruments at follow-up which may have also contributed to the modest follow-up rate and lack of long-term follow-up.

Second, some medical students are keener to learn than others and view any sort of training and assessment as valuable to their career as a future doctor. Additionally some medical students may be more interested in the topic of mental illness related stigma. Given that psychiatry and mental illness are often stigmatised, the topic may have not been interesting to other students thereby leading to their non-participation in the follow-up or study overall.

One might surmise that our lack of results may have been because our participants were already more favourably inclined in terms of attitudes and the intervention more likely to have an effect on those who did not participate. Students knew that the training to be evaluated was part of a research project and that they would not be examined on the material of the training. If the training material and the evaluation were formally embedded in the curriculum this may have led to more participants and possibly more observed effectiveness. Much higher rates have been observed in other settings where this was the case [24].

Information about student enrolment as well as student contact information was provided by the registrar therefore there may have been discrepancies in the actual number of students who were eligible as many students still registered during the two week introductory session and not all students may have informed the registrar if they did not attend the two-week introductory session which was recommended but not compulsory. We were conservative in our response rate estimate since the denominator may have been smaller than that used.

It must be noted that our results may be biased given the students were not a representative sample. Participants were different than non-participants which could have led to biased results. For example, we may have not seen a change in attitudes because participants were already inclined to have positive attitudes. Further, the change in knowledge may have been detected because participants were interested in the material. It is also important to note that the results found may also be due to the intervention being very short in duration as the lecture was one hour and the role-play training was 30 minutes while for medical students, a longer intervention may be required however this would need to be determined in a larger, representative sample.

### Effectiveness of training

Despite the relatively small sample sizes and limited time for intervention, change was detected in knowledge in medical students who received the experimental conditions. We can be confident that the training was successful in leading to greater knowledge amongst these students than those in the control condition. Changes in attitudes were promising and warrant further investigation but not strong enough to be conclusively demonstrated. There was no evidence that behaviour changed.

This was the first trial with two experimental conditions to examine knowledge, attitudes and behaviour after mental illness related stigma training in medical students. Previous studies have either looked at changes in attitudes after psychiatric clerkships or before or after mental illness related stigma training without a behavioural component [25-27].

## Conclusions

A lecture containing personal testimonies of service users and caregivers alone was effective in improving medical students knowledge of the stigma and discrimination issues of people with mental illness but the role-play training was not beneficial in further enhancing knowledge in the short term. The evidence for these interventions in improving attitudes and behaviour was weak and should be interpreted with caution. This finding contributes to the knowledge, attitudes and behaviour (skills) paradigm applied in medical education as well as health promotion. Logistical and implementation lessons from this trial suggest that both training and evaluation elements, including assessing knowledge, attitudes and behaviour, need be embedded in medical students curriculum and assessments to widen the impact of these for medical students. Further evaluation of whether knowledge gains are maintained in the long-term, the required "intensity" and delivery of intervention [34] and more sophisticated assessment of behavioural change should be the next steps in the research agenda. Given very recent research showing that people with mental illness from the same locale as this intervention study lose on average between 8 and 17.5 years of life through earlier mortality [35] and that access to life saving interventions is poorer for those with mental illness [16] such educational initiatives would seem a prerequisite of good medical education.

## Competing interests

The authors declare that they have no competing interests.

## Authors' contributions

AK carried out the coordination, design and implementation of the trial. NG supervised AK and provided guidance on the study methodology. JL was responsible for coordination of the training interventions. ML participated in the design of the study and performed the statistical analysis. GT participated in the study design and coordination and helped to draft the manuscript. All authors read and approved the final manuscript.

## Pre-publication history

The pre-publication history for this paper can be accessed here:

http://www.biomedcentral.com/1472-6920/11/51/prepub
